# Clinical perspectives on the use of the GIP/GLP-1 receptor agonist tirzepatide for the treatment of type-2 diabetes and obesity

**DOI:** 10.3389/fendo.2022.1004044

**Published:** 2022-10-13

**Authors:** Baptist Gallwitz

**Affiliations:** Department of Medicine IV (Diabetology, Endocrinology and Nephrology), Tübingen University, Tübingen, Germany

**Keywords:** glucagon-like peptide-1 receptor agonist (GLP-1RA), incretin based therapy, glucose-depent insulinotropic peptide (GIP), dual agonists, tirzepatide, type 2 diabetes

## Abstract

Incretin-based therapies with glucagon-like peptide-1 receptor agonists (GLP-1RA) are already established in the treatment of type 2 diabetes (T2D). The development of novel dual- or triple-receptor agonists that bind to the receptors not only for GLP-1 but also to the receptors for glucose-dependent insulinotropic polypeptide (GIP) and/or glucagon is intended to address different metabolic pathways for carbohydrate, lipid, and protein metabolism simultaneously. Dual- and triple-receptor agonists acting *via* different receptors and postreceptor pathways seem attractive in view of potentially additive or synergistic effects in the treatment of T2D and obesity. Recently, the first approval for a dual-receptor agonist marks an important step in this development. The GIP/GLP-1-receptor agonist tirzepatide was approved for the treatment of T2D by the Food and Drug Administration (FDA) in the USA for once-weekly subcutaneous injections in May 2022 and has just received a positive opinion from the European Medicines Agency (EMA). Tirzepatide dose-dependently leads to clinically significant reductions in glycemic parameters and body weight and has been shown to have stronger effects in reducing these parameters than standard antidiabetic therapy. This article summarizes the current clinical study program and the respective outcomes and highlights further potential indications for tirzepatide in the treatment of obesity and potentially other comorbidities of T2D.

## Introduction

Recent data from the International Diabetes Foundation (IDF) Diabetes World Atlas released at the end of 2021 shows a consistently rising prevalence of type 2 diabetes (T2D) with different incidence increases in various regions of the world. The global prevalence of 537 million affected in the age group of 20–79 years in 2021 will rise to 783 million in 2045, with the lowest increase in incidence in Europe (13%) and the highest in the African countries south of the Sahara (134%) (1). Diabetes accounts for premature mortality and a loss of life expectancy of approximately 6 years due to vascular comorbidities and complications when glucose control is suboptimal (2). Randomized prospective studies have demonstrated a significant reduction in microvascular and macrovascular complications in patients with T2D when plasma glucose, blood pressure, and plasma lipid concentrations are lowered towards a normal level (3–15). In the past years, therapy for T2D has changed goals from a predominantly “glucocentric” approach to a “patient-centered,” individualized approach taking the patient’s characteristics and comorbidities more into focus for treatment. This approach has initially been promoted by the joint recommendations for a novel treatment algorithm for T2D by the American (ADA) and European Diabetes Associations (EASD) in 2018 that is continuously being updated (16, 17).

Obesity, defined as a body mass index above 30 kg/m^2^, is also increasingly prevalent worldwide and has become a challenge to the various healthcare systems and societies. The prevalence of this condition has nearly tripled in the time span between 1975 and 2016. In 2016, 650 million people were obese and in 2020, 39 million children under the age of 5 were already overweight or obese (18). Obesity is not only a major risk factor for the development of T2D but also a risk factor for developing cardiovascular disease, sleep apnea and chronic pulmonary disease, various cancer manifestations, osteoarthritis, reflux disease, gallstones, nonalcoholic steatohepatitis (NASH), and depression (18–22).

Current guidelines for the treatment of diabetes and obesity recommend a step-wise approach starting with lifestyle interventions aimed at body weight loss, an increase in physical activity, and additional measures for healthier choices in everyday decisions and habits. The lifestyle interventions should be monitored, adapted, and perpetuated in an “informed consent” with the patient over the years, regardless of whether additional medical or surgical therapy is started in the course of T2D or obesity (16, 17, 23–25).

## Incretin-based therapies

Incretins are hormones that are secreted from enteroendocrine cells in the gut mucosa after a meal. They are potent stimulators of postprandial insulin secretion under hyperglycemic conditions and contribute to approximately 70% of the physiological postprandial insulin secretion. The so-called incretin effect describes this phenomenon and also explains that orally ingested glucose leads to a higher insulin response than intravenously administered glucose leading to isoglycemic glucose excursions compared to oral glucose (26, 27). In man, the peptides glucagon-like peptide-1 (GLP-1) and glucose-dependent insulinotropic polypeptide (GIP) are the major incretin hormones. While GIP loses its insulinotropic effect in T2D with chronic hyperglycemia, GLP-1 is still able to stimulate insulin secretion (28, 29). Parenteral administration of pharmacological doses of GLP-1 is able to normalize plasma glucose by stimulating insulin secretion and simultaneously inhibiting glucagon secretion in subjects with T2D and hyperglycemia (30). Native GLP-1 is not suitable for treatment, since the peptide is degraded rapidly within minutes by the enzyme dipeptidyl-peptidase-IV (DPP-4) (31, 32). Utilizing the concept of elevating GLP-1 concentrations, DPP-4 inhibitors as oral agents and synthetic injectable, DPP-4 resistant GLP-1 receptor agonists (GLP-1RA) were introduced as treatment options for T2D therapy (31–36). GLP-1RA provides strong and effective glycemic control and also lower body weight and systolic blood pressure. They have a low risk of causing hypoglycemia, and they also promote satiety by central nervous effects and by slowing gastric emptying.

There are, however, challenges and limitations regarding therapy with GLP-1RA. Gastrointestinal side effects, mostly with symptoms of transient fullness and nausea, are typical for the initiation of treatment. They affect 20%–30% of patients and usually cease during long-term therapy after a few weeks. Therapy with GLP-1RA should therefore start with low doses that can be uptitrated (33, 34). Generally, the gastrointestinal side effects are more pronounced with shorter-acting GLP-1RA in comparison to long-acting compounds. The individual tolerability and severity of gastrointestinal symptoms at the beginning of therapy are heterogeneous (36). GLP-1RA should not be used in patients with a history of acute or chronic pancreatitis and therapy should be stopped immediately if there are clinical signs and symptoms of acute pancreatitis. A symptom-free elevation in plasma amylase and/or lipase activity is commonly observed with GLP-1 RAs. In these cases, therapy can be continued. Another contraindication is the use of GLP-1RA in patients with a history of medullary thyroid carcinoma or multiple endocrine neoplasia type 2 (MEN2 (33, 34, 36). With the exception of the oral formulation of semaglutide, GLP-1RAs have to be injected subcutaneously daily or once weekly (33, 34, 36).

GLP-1RA has been placed prominently in the international recommendations for the treatment of T2D as the first injectable agent before insulin due to the advantageous outcomes in cardiovascular safety studies that demonstrated a reduction of the combined cardiovascular endpoint three-component major adverse cardiovascular event (MACE-3; cardiovascular death, nonfatal myocardial infarct, nonfatal stroke) for the GLP-1RA albiglutide, efpeglenatide, dulaglutide, liraglutide, and semaglutide (10–18).

The GLP-1RA liraglutide has also been approved for the treatment of obesity at a higher standard dose (3 mg once daily) compared to the standard dose used for T2D treatment (1.2 mg once daily) due to the results of the SCALE study program (37, 38).

## Development of dual- and triple-receptor agonists

The pathophysiology of T2D and obesity is complex and heterogenous on an individual level. Therefore, therapeutic principles acting *via* different receptors and postreceptor pathways seem attractive in view of potentially additive or synergistic effects. Following this strategy, different metabolic pathways for carbohydrate, lipid, and protein metabolism could be influenced simultaneously. Likewise, energy metabolism and appetite regulation could be altered in a therapeutic direction (39–41).

### The rationale for dual-receptor agonists for GLP-1 and GIP

**Figure 1:**
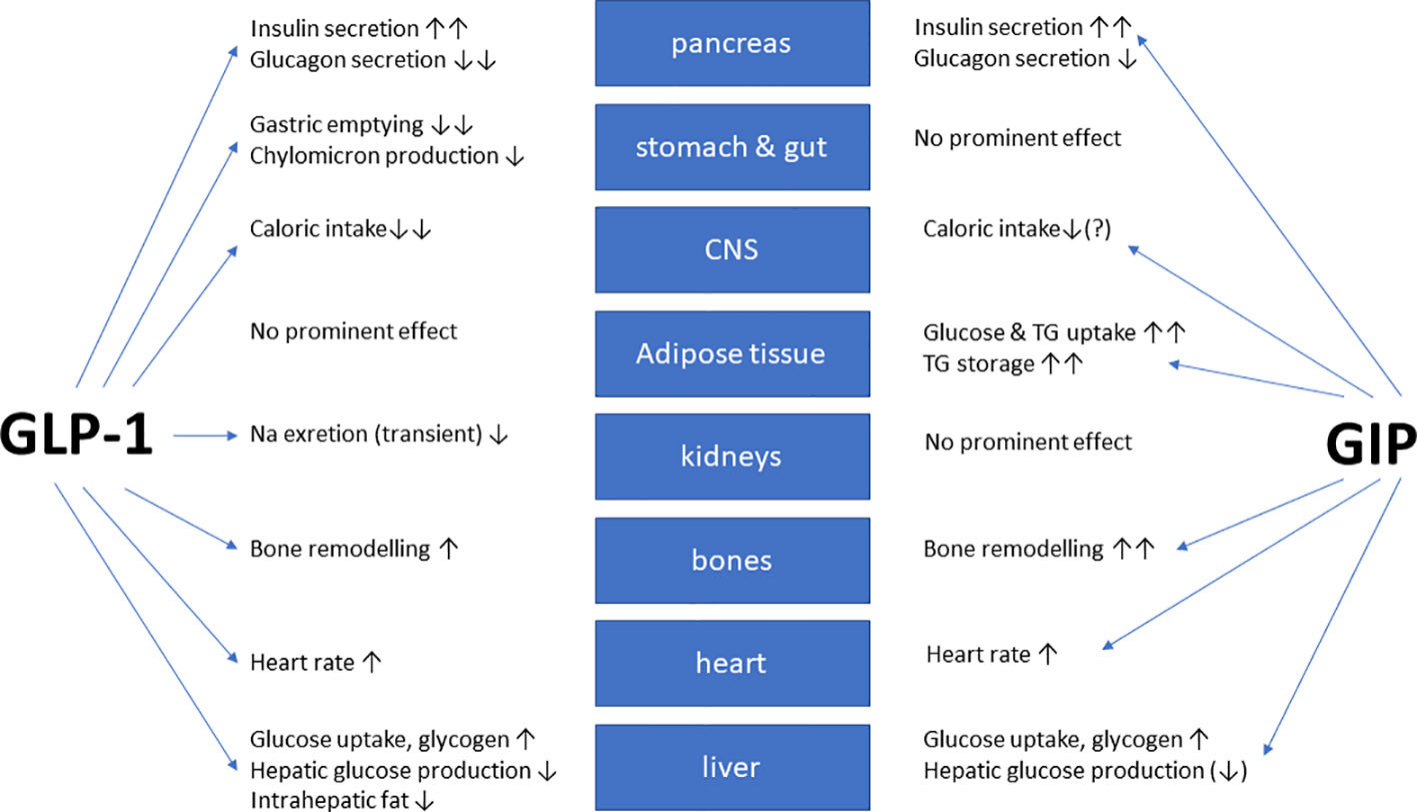
Overview of the biological glucose-dependent insulinotropic polypeptide (GIP) and glucagon-like peptide-1 (GLP-1) effects at the organ/tissue level (modified from (47)). The blue arrows depict relevant physiological effects.

In healthy volunteers, infusions of GLP-1 and GIP had additive effects on the stimulation of insulin secretion (42). The peptides GLP-1 and GIP have a high amino-acid sequence similarity in the N-terminal part of the peptide. They bind to highly selective specific receptors, GLP-1 has a very low affinity towards the GIP receptor and vice versa (43). *In vitro* receptor binding studies explored structural requirements for GLP-1 and GLP-1/GIP chimeric peptides regarding their affinity towards the GLP-1 receptor in insulinoma cell lines (44, 45). While GLP-1 also inhibits appetite and food intake, GIP apparently does not have such effects. On the contrary, many studies have even suggested that GIP may promote obesity. Due to these findings, GIP did not seem to be a therapeutic option for T2D or obesity (46, 47). **Figure 1** gives an overview of the physiological actions of GLP-1 and GIP on various organ systems (47). GLP-1/GIP chimeric peptides capable of activating both receptors have shown remarkable weight-losing and glucose-lowering efficacy in obese individuals with T2D. Likewise, GIP receptor antagonists have been reported to induce weight loss in animal studies. Therefore, both agonists and antagonists of the GIP receptor may be useful for the therapy of obesity (46). The exact mechanisms explaining these findings are not completely understood, but there is some evidence that agonist-induced internalization of the two receptors for GLP-1 and GIP differs markedly. Structural alterations of the ligand peptides as in GIP/GLP-1 dual-receptor agonists may alter these cellular processes strongly and may explain that an antagonist may activate while an agonist may block receptor signaling (46–49). The most important known patterns for the therapeutic effects of GIP agonism as well as antagonism in preventing obesity and in reducing body weight are summarized in **Table 1** (49).

**Table 1:** Effects of GIP receptor agonism or antagonism on glycemic control, body weight, and energy balance in animal models of obesity and diabetes (modified according to Campbell and Nauck) (47, 49).

Experimental approach		Glycemic homeostasis		Body weight/energy household		
Intervention regarding GIP receptor stimulation	Model	Glucose tolerance	Insulin resistance	Body weight	Energy intake	Energy expenditure
Antagonism	Prevention of diet-induced obesity and diabetes	↑	↓	↓↓	(↓)	0
	Treatment of pre-existent obesity and diabetes	↑↑	↓	(↓)	0-(↓)	0
Agonism	Prevention of diet-induced obesity and diabetes	0	0	0	0-(↓)	0
	Treatment of pre-existent obesity and diabetes	↑↑	(↓)	0-(↓)	0-(↓)	0

Prevention of diet-induced obesity: healthy, nonobese animals at baseline receiving experimental treatment while receiving a high-fat diet. Treatment of pre-existing obesity: animals with genetic mutations (ob/ob or db/db mice) causing obesity or high-fat diet-induced obesity at baseline. Agonism summarizes peptide GIP agonists, interventions leading to GIP hypersecretion, or antibody-mediated stimulation of GIP receptors; antagonism summarizes peptide GIP antagonists, interventions against K cells or GIP secretion, or specific antibodies either inactivating circulating GIP or GIP receptors. “(↑), ↑, ↑↑” trend or significant increment in this parameter (weak, intermediate, or strong effect); “(↓), ↓, ↓↓” trend or significant reduction in this parameter (weak, intermediate, or strong effect); 0, no obvious effect. Only patterns that are representative of all published studies in this category have contributed to the conclusions summarized in this table.

### Dual-receptor agonists for GLP-1 and glucagon

Glucagon and GLP-1 also share sequence similarities in their peptide chains. Glucagon is also involved in acutely regulating glucose homeostasis and administration of pharmacological doses of glucagon in animal models for diabetes or obesity has demonstrated regulatory effects on lipid metabolism, energy expenditure, and food intake (41, 50). On the basis of these findings, dual-receptor agonists for GLP-1/GIP, GLP-1/glucagon as well as triple-receptor agonists were propagated and successively developed for the treatment of T2D and obesity (39–41, 51).

The GLP-1/glucagon agonist cotadutide (MEDI0382) is the compound of this dual-receptor agonist class that is the most advanced in clinical development. In preclinical studies, cotadutide demonstrated a more pronounced loss of body weight, reduction in food intake, and superior improvement in glycemic parameters in rodents compared to a GLP-1 RA. Clinical studies showed an improvement in glycemic parameters and a substantial and dose-dependent loss of body weight. A daily dose of 300 µg of codatudide resulted in a significantly greater loss of body weight compared to 1.8 mg of liraglutide given once daily. Lipids and liver enzymes were also lowered under codatudide administration. At present, studies are ongoing to investigate the influence of this dual-receptor agonist on parameters for NASH and liver fibrosis. Gastrointestinal side effects observed under cotadutide effects are similar in quality but more severe than those under GLP-1RA treatment (52).

Animal data on a GLP-1/GIP/glucagon triple agonist show strong effects on body weight reduction, and glucose- and lipid normalization in rodents. Clinical studies in humans have not been published so far (41, 51).

## Development, preclinical, and early clinical data on the GIP/GLP-1 receptor agonist tirzepatide

Among the GIP/GLP-1 receptor agonists, tirzepatide (LY3298176) is the compound that is the most advanced in development and the first substance in this class that has received approval for the therapy of T2D by the FDA in May 2022 (manufacturer Eli Lilly Comp., trade name Mounjaro^®^) (53–55). In July 2022, the EMA adopted a positive opinion, recommending the granting of marketing authorization for tirzepatide as a therapeutic agent for T2D (56).

### Pharmacodynamics

**Figure 2:**
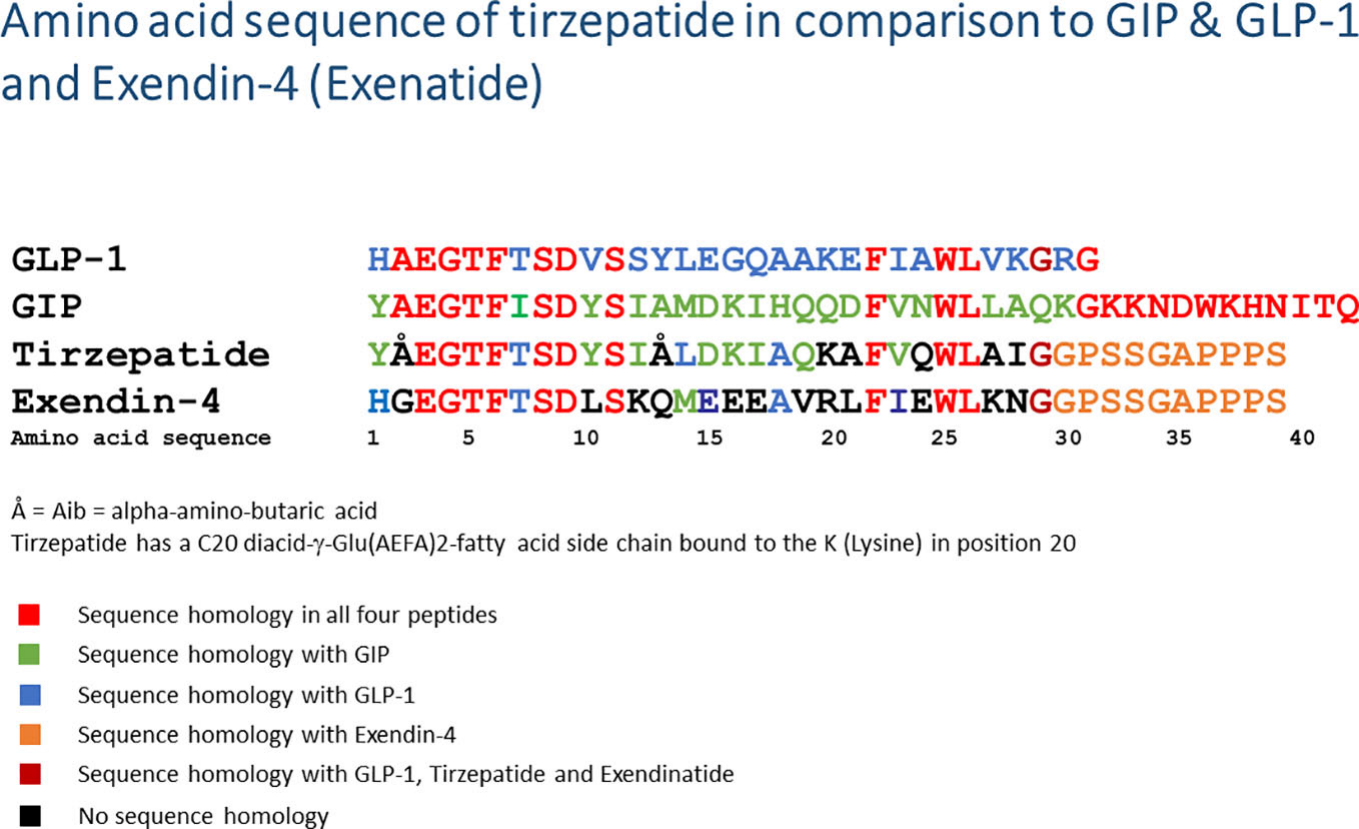
Amino acid sequence of tirzepatide in comparison to native GIP, GLP-1, and exendin-4 (exenatide). The amino acid sequences of the peptides are given in a one-letter code starting with the N-terminus on the left side. Å = Aib, alpha-amino-butyric acid; diacid-γ-Glu(AEFA)2, the linker molecule linking a fatty acid side chain with a length of 20 carbon atoms (=C20) to the K (lysine) in position 20; GLP-1, glucagon-like peptide-1; GIP, glucose-dependent insulinotropic polypeptide.

Tirzepatide is a GIP/GLP-1 chimeric peptide with a peptide chain length of 39 amino acids. The linear peptide is covalently bound to a C20 fatty diacid moiety at the side chain of the Lys20 amino acid (57). **Figure 2** depicts the amino acid sequence of tirzepatide in comparison to GLP-1, GIP as well as the GLP-1RA exendin-4 (exenatide). Comparable to other peptides linked to fatty acids (e.g., the insulins detemir and degludec, the GLP-1RA liraglutide and semaglutide), the fatty acid side chain allows binding to albumin after subcutaneous injection, slows enzymatic degradation and prolongs biological half-life (57–60). Tirzepatide binds with high affinity to both receptors, GIP (Ki = 0.135, similar to native GIP) and GLP-1 (Ki = 4.23, appr. 5-fold weaker affinity than native GLP-1). *In vitro* signaling studies demonstrated that tirzepatide was able to activate the GIP receptor with comparable potency to native GIP (57). The activating potency of the GLP-1 receptor was also demonstrated, but this was 13-fold weaker compared to native GLP-1. Compared to the GLP-1RA semaglutide, tirzepatide was a less potent GLP-1RA. In all *in vitro* and *in vivo* models used, tirzepatide was able to stimulate glucose-dependent insulin secretion *via* GIP and/or GLP-1 receptor activation. In a rodent model for obesity, tirzepatide administration induced a loss of body weight, by mechanisms of reducing food intake and by increasing energy expenditure (57). A phase Ib trial with a 4-week subcutaneous administration of either 10 mg or 15 mg tirzepatide once weekly in subjects with T2D led to a significant reduction in fasting plasma glucose concentrations (LSM difference [95% CI]: −49.12 mg/dl [−78.14, −20.12] and −43.15 mg/dl [−73.06, −13.21], respectively) and to reductions in body weight (LSM difference [95% CI]: −2.62 kg [−3.79, −1.45] and −2.07 kg [−3.25, −0.88], respectively; clinical trial registration number NCT02759107) (57). Likewise, first- and second-phase insulin secretion and insulin sensitivity were increased, while postprandial plasma glucose concentrations and plasma glucagon concentrations were lowered. Gastric emptying was delayed, similarly to the effect observed under therapy with GLP-1RA (61). The data on insulin secretion, insulin sensitivity, and glucose homeostasis were confirmed in the phase II clinical study (clinical trials registration number NCT03131687) (62). In the phase II program, tirzepatide was also shown to improve biomarkers for cardiovascular risk and to improve NASH (63–65).

### Pharmacokinetics

The pharmacokinetics in healthy volunteers and patients with T2D are comparable (57). Tirzepatide exposure increases dose-proportionally after subcutaneous injection irrespective of the injection site (61). The time to maximum plasma concentration (tmax) spans from 8 to 72 h. The bioavailability amounts to 80%. A steady state in plasma concentrations is achieved after four once-weekly injections. Approximately 99% of the bioavailable tirzepatide is bound to plasma albumin. The metabolic clearance occurs by proteolytic cleavage of the tirzepatide peptide backbone, β-oxidation of the C20 fatty diacid moiety, and by amide hydrolysis. The metabolites are excreted *via* the urine and feces. The half-life amounts to approximately 5 days. These pharmacokinetic data allow a once-weekly subcutaneous injection regimen. The pharmacokinetics of tirzepatide are not significantly changed by age, gender, race, ethnicity, body weight, or renal or hepatic function (61). CYP enzyme activity is not influenced by tirzepatide according to the presently available study data and drug transporters are not inhibited (61). The absorption of concomitant oral medications may be reduced or delayed when tirzepatide therapy is initiated; this effect is explained by the delay of gastric emptying observed under tirzepatide administration (61). This effect is relevant for women using oral contraceptives. Women should not only rely on oral contraception in such a situation but either add a barrier contraception method for 4 weeks after initiation until four 4 weeks after the last dose escalation of tirzepatide or switch to a completely nonoral contraceptive method (61).

## Clinical data on tirzepatide in type 2 diabetes—the SURPASS clinical trial program

Early clinical studies with tirzepatide revealed a marked and dose-dependent reduction of HbA1c and further glycemic parameters in patients with T2D in various ethnic cohorts (clinical trials registration numbers NCT03322631, NCT03131687, and NCT03311724) (66–68). A phase II dose-finding study found that tirzepatide (1, 5, 10, and 15 mg once weekly) was noninferior at doses >5 mg once weekly in reducing HbA1c (primary endpoint) and body weight (secondary endpoint) in comparison to dulaglutide (1.5 mg once weekly) (66). Comparable to therapy with GLP-1RA, a stepwise dose escalation starting with a low starting dose was associated with a lower rate of gastrointestinal side effects and better tolerability of tirzepatide (67).

For the phase III clinical program, a total of eight studies were initiated that investigated the efficacy and safety of tirzepatide in comparison to various other established antidiabetic medications in T2D either in monotherapy, combination with metformin as baseline therapy, or metformin and insulin glargine as a baseline. The design of these studies was comparable regarding the tirzepatide doses, starting with 2.5 mg once weekly with dose increases by increments of 2.5 mg every 4 weeks until the randomized final treatment dosages of 5, 10, or 15 mg tirzepatide were reached. This study program named SURPASS comprises six global, two Japanese, and one Asian-Pacific study (69). **Table 2** gives an overview of the characteristics and results of the entire SURPASS study program (69–74). The study results of the studies SURPASS-1 to SURPASS-5 have been published, and the remaining studies (SURPASS-6, SURPASS-CVOT, SURPASS-J-mono, SURPASS-J-combo; SURPASS-AP-combo, respective clinical trial registration numbers NCT04537923, NCT04255433, NCT03861052, NCT03861039, NCT04093752) are not completed yet.

**Table 2:** Overview of the SURPASS clinical trial program with tirzepatide in the therapy of type 2 diabetes.

Study	Subjects (*n*)	Baseline therapy	Diabetes treatment in the comparator arm	Study duration (weeks)	HbA1c lowering *vs*. comparator (%)	Body weight change (kg)	Publication/anticipated end of the trial
SURPASS-1	478	None	Placebo	40	BL: 7.9 5 mg: −1.91 10 mg: −1.93 15 mg: −2.11	−7.0 to −9.5	Rosenstock et al. Lancet 2021;398:143–155 (70)
SURPASS-2	1,879	Metformin	Semaglutide 1 mg	40	BL: 8.28 5 mg: −2.01 10 mg: −2.24 15 mg: −2.30 Semaglutide: −1.87	5 mg: −1.9 10 mg: −3.6 15 mg: −5.5	Frias et al. NEJM 2021;385:503–515 (71)
SURPASS-3	1,437	Metformin or metformin + SGLT-2i	Insulin degludec	52	BL: 8.17 5 mg: −1.93 10 mg: −2.20 15 mg: −2.37 Insulin degludec: 1.34	−7,5 to −12.9 Insulin degludec: +2.3	Ludvik et al. Lancet 2021;398:583–598 (72)
SURPASS-4	1,995	1-3 OAD with metformin, SGLT-2i, or SU	Insulin glargine	52	BL: 8.52 5 mg: −2.24 10 mg: 2.43 15 mg: 2.58 Insulin glargine: 1.44		Del Prato et al. Lancet 2021; 398:1811–1824 (73)
SURPASS-5	475	Insulin glargine ± metformin	Placebo	40	BL: 8.31 5 mg: 2.11 10 mg: 2.40 15 mg: 2.34		Dahl et al. JAMA 2022;327:534–545 (75)
SURPASS-6	1,182	Insulin glargine ± metformin	Insulin lispro	52			Estimated study end Q2 2022
SURPASS-J mono	636	Therapy naive or 1 OAD	Dulaglutide 0.75 mg	52			Study finished, results not yet published
SURPASS-J combo	441	1 OAD	No comparator arm (safety study, endpoint >1 SAE)	52			Study finished, results not yet published
SURPASS AP combo	956	Metformin ± SU	Insulin glargine	40			Study recruitment completed, study still ongoing
SURPASS CVOT	12,500	OAD or injectable antidiabetic medication	Dulaglutide 1.5 mg	Event driven regarding MACE-3			Estimated study end 2024/25

SGLT-2i, SGLT-2 inhibitor; OAD, oral antidiabetic drug; SU, sulfonylurea; SAE, serious adverse event; MACE-3, major cardiovascular event (cardiovascular death, nonfatal myocardial infarct, or nonfatal stroke); BL, baseline.

The SURPASS-1 trial investigated the efficacy and safety of the three doses of 5, 10, or 15 mg tirzepatide in drug-naïve patients with T2D who were not sufficiently controlled with a lifestyle intervention with diet and exercise versus placebo (70). A total of 478 patients were randomized into equally sized four study arms. The baseline HbA1c in the tirzepatide arms was 7.85%–7.97% and 8.05% in the study arm receiving a placebo. For the efficacy estimand, the LSM change in HbA1c from baseline at week 40 as the primary endpoint was −1.87%, −1.89%, and −2.07% with tirzepatide 5, 10, and 15 mg, respectively, versus +0.04% with placebo (*p* < 0.0001 for all tirzepatide arms *vs*. placebo). Significantly more patients on tirzepatide (*p* < 0.0001) reached an HbA1c of <7% at week 40 (87%–92% *vs*. 19%). The LSM change in body weight from baseline at week 40 was −7.0, −7.8, and −9.5 kg with tirzepatide 5, 10, and 15 mg, respectively, versus −0.7 kg with placebo (*p* < 0.0001 for all tirzepatide arms *vs*. placebo). Mild to moderate transient gastrointestinal side effects were the most frequently reported adverse events associated with tirzepatide treatment (nausea 12%–18% *vs*. 6%; diarrhea 12%–14% *vs*. 8%; vomiting 2%–6% *vs*. 2%). No clinically significant or severe hypoglycemic incidents were reported with tirzepatide. Tirzepatide demonstrated significant reductions in HbA1c and body weight without an increased risk of hypoglycemia. Tirzepatide has a comparable safety profile to GLP-1RA (54, 70).

In the SURPASS-2 study, the three doses of tirzepatide (5, 10, and 15 mg once weekly) were tested with the GLP-1RA semaglutide (1 mg once weekly) as a comparator in patients not optimally controlled on monotherapy with metformin (71). In this study, 1,879 patients were randomized. The baseline HbA1c in the tirzepatide arms was 8.26%–8.32% and 8.25% in the study arm receiving a placebo. For the efficacy estimand, the LSM change in HbA1c from baseline at week 40 as the primary endpoint was −2.01%, −2.24%, and −2.30% with tirzepatide 5, 10, and 15 mg, respectively, versus −1.86% with semaglutide (*p* < 0.02 for all tirzepatide arms *vs*. semaglutide). Regarding the primary endpoint, all doses of tirzepatide were superior in comparison to semaglutide. Body weight loss was more pronounced with tirzepatide than with semaglutide (LSM estimated treatment difference, −1.9, −3.6, and −5.5 kg, respectively; *p* < 0.001 for all comparisons). Mild to moderate transient gastrointestinal side effects were the most frequently reported adverse events associated with tirzepatide treatment and were similar in frequency and intensity compared to semaglutide (nausea 17%–22% *vs*. 18%; diarrhea 13%–16% *vs*. 12%; vomiting 6%–10% *vs*. 8%). Nonsevere, hypoglycemia (blood glucose, <54 mg/dl) occurred with a low rate in 0.6%, 0.2%, and 1.7% (in the 5-, 10-, and 15-mg tripeptide arms, respectively), and in 0.4% in the semaglutide group. In summary, tirzepatide was noninferior and superior to semaglutide with respect to the reduction of HbA1c from baseline to 40 weeks (54, 71).

The SURPASS-3 study was set up to investigate the efficacy and safety of tirzepatide versus a titration of the basal insulin degludec inadequately controlled by an oral therapy of T2D by metformin with or without an SGLT2 inhibitor comedication for 52 weeks (72). A total of 1437 patients were randomized. In the insulin comparator arm, insulin degludec was given once daily at bedtime and titrated to morning fasting blood glucose of <90 mg/dl. The baseline HbA1c in the tirzepatide arms was 8.17%–8.21% and 8.12% in the study arm receiving insulin degludec. For the efficacy estimand, the LSM change in HbA1c from baseline at week 52 as the primary endpoint was −1.93%, −2.20%, and −2.37% with tirzepatide 5, 10, and 15 mg, respectively, versus −1.34% with insulin degludec (*p* < 0.02 for all tirzepatide arms *vs*. semaglutide). Tirzepatide at the doses of 10 and 15 mg was noninferior to insulin degludec (primary endpoint) and all three doses of tirzepatide demonstrated superiority in comparison to insulin degludec regarding the change in HbA1c. Significantly more study participants using tirzepatide reached an HbA1c <7% at week 52 (82%–93% *vs*. 61% for insulin degludec; *p* < 0.0001) The LSM change in body weight from baseline at week 52 was −7.5, −10.7, and −12.9 kg with tirzepatide 5, 10, and 15 mg, respectively, versus +.3 kg with insulin degludec (*p* < 0.0001 *vs*. insulin degludec for all). Hypoglycemia (<54 mg/dl or severe) was reported in five (1%), four (1%), and eight (2%) participants on tirzepatide 5, 10, and 15 mg, respectively, versus 26 (7%) on insulin degludec (54, 72). A substudy of SURPASS-3 investigated glycemia using continuous glucose monitoring (CGM) (73). In 243 patients of the SURPASS-3 trial using CGM, the proportion of time within a tight predefined “time in range” (TIR) of 71–140 mg/dl at the study end was significantly (*p* < 0.0001) greater in the pooled group of participants receiving either 10 or 15 mg tirzepatide compared to the group receiving insulin degludec (73% *vs*. 48%; primary endpoint) (54, 73).

The efficacy and safety of tirzepatide, with a special focus on cardiovascular safety, were investigated in the 52-week SURPASS-4 trial testing tirzepatide versus insulin glargine in adults with T2D and a high cardiovascular risk under inadequate glycemic control at baseline on one to three oral glucose-lowering medications (metformin, sulfonylurea, SGLT-2 inhibitor) (54, 74). In this study, 2,002 patients were randomized. In the insulin comparator arm, insulin glargine was given once daily at bedtime and titrated to morning fasting blood glucose of <100 mg/dl. The baseline HbA1c in the tirzepatide arms was 8.52%–8.59% and 8.50% in the study arm receiving insulin glargine. For the efficacy estimand, the LSM change in HbA1c from baseline at week 52 as the primary endpoint was −2.24%, −2.43%, and −2.58% with tirzepatide 5, 10, and 15 mg, respectively, versus −1.44% with insulin glargine. The higher tirzepatide doses of 10 and 15 mg were noninferior compared to insulin glargine for the change in HbA1c (primary endpoint). Significantly more study participants using tirzepatide reached an HbA1c of <7% at week 52 (81%–91% *vs*. 51% for insulin glargine; *p* < 0.0001). The LSM change in body weight from baseline at week 52 was −7.1, −9.5, and −11.7 kg with tirzepatide 5, 10, and 15 mg, respectively, versus +1.9 kg with insulin glargine (*p* < 0.0001 *vs*. insulin glargine for all). Gastrointestinal adverse effects in the tirzepatide groups were similar to those in the other SURPASS trials. Only 6%–9% of study participants on tirzepatide experienced hypoglycemia (glucose <54 mg/dl or severe) compared to 19% in the insulin glargine group. Particularly, participants not receiving sulfonylureas as baseline therapy profited from tirzepatide regarding hypoglycemic episodes (tirzepatide 1%–3% *vs*. glargine 16%). Adjudicated four-component major adverse cardiovascular event (MACE-4 events; cardiovascular death, myocardial infarction, stroke, hospitalization for unstable angina) occurred in 109 participants. The hazard ratio and 95% confidence interval did not reveal a significant difference between tirzepatide or insulin glargine treatment (HR, 0.74; 95% CI, 0.51–1.08). Deaths (*n* = 60) in total occurred in the study, 25 (3%) in the tirzepatide arms and five (4%) in the insulin glargine arm (HR, 0.70; 95% CI, 0.42–1.17). For the four study arms, the occurrence of death was 15 (5%) for the tirzepatide 5 mg dose, two (<15) for the tirzepatide 10 mg dose, eight (2%) for the tirzepatide 15 mg dose, and 25 (3%) for all tirzepatide doses versus 35 (4%) for the insulin glargine arm. None of the deaths were considered to be related to study medication. Six deaths (<1%) in participants receiving tirzepatide and nine (<1%) in participants receiving glargine were adjudicated as cardiovascular. Ten deaths (1%) on tirzepatide and 12 (1%) on glargine were adjudicated as undetermined, and thus considered cardiovascular deaths for MACE determination. Six COVID-19–related deaths were recorded in participants treated with tirzepatide (<1%) and eight (<1%) on glargine (74). In summary, tirzepatide achieved more pronounced HbA1c reductions with a lower incidence of hypoglycemia at week 52 compared to insulin glargine. Tirzepatide treatment was not associated with excess cardiovascular risk (54, 74).

The SURPASS-5 study assessed the efficacy and safety of an injectable combination therapy combining tirzepatide with insulin glargine (75). Patients with T2D not adequately controlled on therapy with metformin and insulin glargine as baseline therapy received either tirzepatide at the doses used in the SURPASS trials or placebo during the 40-week study setting. In this study, 475 patients were randomized. The baseline HbA1c in the tirzepatide arms was 8.23%–8.36% and 8.37% in the placebo group. For the treatment-regimen estimand, the LSM change in HbA1c from baseline at week 40 as the primary endpoint was −2.40% and −2.34% with tirzepatide 10 and 15 mg, respectively, versus −0.86% with placebo (*p* < 0.001 *vs*. placebo for both; primary endpoint). The higher tirzepatide doses of 10 and 15 mg were noninferior compared to insulin glargine for the change in HbA1c (primary endpoint). Significantly, more study participants using tirzepatide reached an HbA1c of <7% at week 40 (85%–90% *vs*. 35% for placebo, *p* < 0.001). The LSM change in body weight from baseline at week 52 was −5.4, −7.5, and −8.8 kg with tirzepatide 5, 10, and 15 mg, respectively, versus +1.6 kg with placebo (*p* < 0.001 *vs*. placebo for all). The investigators conclude that inadequately controlled patients with T2D on metformin and insulin glargine profit from the additional therapy with tirzepatide and reach statistically significant improvements in glycemic control after 40 weeks (54, 75).

Preliminary results from the SURPASS-J-mono study in Japanese patients with T2D who were either therapy naïve or on an antidiabetic monotherapy showed a head-to-head comparison of a tirzepatide treatment compared to therapy with the GLP-1RA dulaglutide at a dose of 0.75 mg (54, 76). A total of 636 patients were randomized into equally sized four study arms. The baseline HbA1c in the tirzepatide arms was 8.18%–8.19% and 8.15% in the study arm receiving dulaglutide. For the efficacy estimand, the LSM change in HbA1c from baseline at week 52 as the primary endpoint was −2.37%, −2.55%, and −2.82% with tirzepatide 5, 10, and 15 mg, respectively, versus −1.29% with dulaglutide (*p* < 0.001 for all tirzepatide arms *vs*. placebo) (54, 76).

## Preliminary cardiovascular outcome data on tirzepatide in type 2 diabetes

The above-mentioned preliminary cardiovascular safety data from the SURPASS-4 study demonstrated no excess cardiovascular risk associated with tirzepatide therapy (54, 73). A recent meta-analysis of data from seven double-blind randomized controlled trials with tirzepatide lasting at least 26 weeks confirmed the cardiovascular safety of tirzepatide in patients with T2D (77). In this meta-analysis, the time span to the first occurrence of a confirmed MACE-4 (cardiovascular death, myocardial infarction, stroke, and hospitalized unstable angina) between study participants with T2D receiving tirzepatide was compared with the control groups receiving different standard therapy. The hazard ratios (HRs) and confidence intervals (CIs) were calculated using a stratified Cox proportional hazards model in which treatment was considered a fixed effect and trial-level cardiovascular risk as the stratification factor. Data from a total of 7,215 study participants were used; the cohort of tirzepatide-treated patients comprised 4,887 subjects, and the control group had a size of 2,328 patients. Subjects (*n* = 142) had at least one MACE-4 event. The majority of patients with a MACE-4 event (*n* = 109) were participants in the SURPASS-4 trial investigating a study population with a high cardiovascular risk. For MACE-4, the HR was 0.80 (95% CI, 0.57–1.11) for tirzepatide versus standard therapy, the HR for cardiovascular death 0.90 (95% CI, 0.50–1.61) and 0.80 (95% CI, 0.51–1.25) for all-cause death. The observed effects were consistent in all investigated subgroups, with a trend toward stronger effects in subjects with T2D and higher cardiovascular risk (77). The results of the large cardiovascular event-driven safety trial comparing tirzepatide with dulaglutide in 12,500 participants in the SURPASS-CVOT study are expected in 2024 (78).

## Data on tirzepatide in fatty liver disease

Within a substudy with 296 participants of the SURPASS-3 trial (SURPASS-3 MRI), changes in liver fat and other fat compartments were monitored during the study, and a subgroup of the tirzepatide cohort was compared to the insulin degludec comparator subgroup (54, 79). The liver fat content (LFC) was comparable in both groups at baseline (15.67% for the tirzepatide group *vs*. 16.58% for the insulin degludec group). The mean absolute change at the end of the study at week 52 was significantly greater in the pooled tirzepatide 10 and 15 mg groups than in the insulin degludec group (−8.09% *vs*. −3.38%; *p* < 0.0001; primary endpoint). The reduction in LFC was significantly correlated (*p* ≤ 0.0006) with baseline LFC (*ρ* = −0.71), reductions in visceral adipose tissue (VAT) (*ρ* = 0.29), reductions in the transaminase liver enzyme ASAT (*ρ* = 0·33), and reductions in body weight (*p* = 0·34) in the tirzepatide groups (54, 79). These metabolic effects of tirzepatide on the liver are currently further investigated in another study (phase II trial) assessing the efficacy and safety of tirzepatide in patients with nonalcoholic steatohepatitis (SYNERGY-NASH, clinical trial registration number NCT04166773) (54, 80).

## Tirzepatide in obesity—the surmount clinical study program

The GLP-1RA liraglutide has been approved for the pharmacological treatment of obesity and various other GLP-1RA may be approved in the future for this indication as well (39–41, 81). Dual- and triple-receptor agonists also offer the potential for the treatment of obesity (39–41). In patients with T2D being overweight or obese, tirzepatide treatment resulted in significant body weight reductions (69–76). On the basis of these findings, a clinical study program was started to investigate the efficacy and safety of tirzepatide in the treatment of obesity and obesity management. The global respective SURMOUNT study program comprises four randomized controlled trials with a study duration of at least 72 weeks using the tirzepatide doses of 10 and 15 mg in the studies SURMOUNT-2 to SURMOUNT-4 and all three doses, including the 5-mg dose in SURMOUNT-1 (82–85). The SURMOUNT-1 study was a placebo-controlled 72-week trial in obese subjects that randomized 2,539 participants without diabetes into four equally large study arms with placebo or one of the three tirzepatide doses (5, 10, or 15 mg once weekly). After a 20-week titration phase, the participants were followed for another 52 weeks. The co-primary endpoints of the study at 72 weeks were the percentage change in weight from baseline and a body weight reduction of 5% or more (82). The tirzepatide doses of 10 and/or 15 mg demonstrated superiority compared to placebo for the coprimary endpoints percentage body weight reductions from baseline at week 72 and the proportion of patients achieving ≥5% body weight reduction at week 72. For the treatment-regimen estimand, the mean body weight reductions from baseline at 72 weeks were 15.0%, 19.5%, and 20.9% in the tirzepatide 5, 10, and 15 mg groups, respectively, versus 3.1% in the placebo group. The proportion of patients achieving ≥5% bodyweight reduction at week 72 was 85%, 89%, and 91% in the tirzepatide 5, 10, and 15 mg groups, respectively, versus 35% in the placebo group (*p* < 0.001 for all comparisons with placebo). Regarding the secondary endpoint of the proportion of patients achieving ≥20% body weight reduction within the study, there was a dose-dependent group size of 50% of the participants receiving the dose of 10 mg tirzepatide and a proportion of 57% in the study arm with 15 mg. Tirzepatide treatment was also associated with a reduction in waist circumference, systolic blood pressure, fasting insulin and plasma lipid concentrations as cardiometabolic risk parameters. Physical function parameters captured by the standardized 36-Item Short Form Health Survey (SF-36) also improved (54, 82).

The other above-mentioned global SURMOUNT studies are still ongoing, in SURMOUNT-2, 938 participants with T2D are enrolled receiving either 10 or 15 mg of tirzepatide. This is a placebo-controlled study with a duration of 72 weeks (83). In SURMOUNT-3, 600 participants are randomized to receive either tirzepatide at the maximally tolerated dose or placebo following a structured intensive 12-week lifestyle intervention. The study period on medication or placebo following the lifestyle intervention is 72 weeks (84). SURMOUNT-4 is a study investigating body weight maintenance. In this study, a total of 600 participants are randomized. All subjects receive tirzepatide at the maximally tolerated dose for 36 weeks. Following this study period, the patients were randomized into two arms, either continuing tirzepatide therapy or continuing with placebo (85). All studies have the percent change from randomization in body weight as the primary endpoint (82–85).

## Adverse events observed with tirzepatide

The adverse events observed in the clinical study program with tirzepatide are similar to those associated with therapy with GLP-1RA. The most frequent adverse events are gastrointestinal side effects that are mild to moderate, dose-dependent, and transient during the first weeks of treatment. The majority of gastrointestinal adverse events occurred during the titration phase of tirzepatide. **Table 3** summarizes the pooled data on the gastrointestinal adverse reactions observed in the SURPASS-1 to SURPASS-5 studies for T2D therapy in comparison to placebo (61). Adverse events with gastrointestinal symptoms were observed in 37.1%–43.6% of patients in the tirzepatide group and 20.4% of patients in the placebo cohort; 3.0%–6.6% and 0.4% of patients in the respective groups discontinued treatment because of these adverse events (54).

**Table 3:** Pooled gastrointestinal adverse events associated with tirzepatide treatment in the SURPASS 1 and SURPASS 5 studies compared to comparator treatment in type 2 diabetes (54, 70, 75).

Symptom	Tirzepatide 5 mg (total *n* = 237; % of subjects)	Tirzepatide 10 mg (total *n* = 240; % of subjects)	Tirzepatide 15 mg (total *n* = 241; % of subjects)	Placebo/comparator (total *n* = 235; % of subjects)
Nausea	12	15	18	4
Diarrhea	12	13	17	9
Decreased appetite	5	10	11	1
Vomiting	5	5	9	2
Constipation	6	6	7	1
Abdominal pain	6	5	5	4

Comparable to observations during treatment with GLP-1RA, symptom-free elevations of pancreatic enzyme concentrations for amylase and lipase were seen in the above-mentioned SURPASS studies (61). As in the case of GLP-1RA, the significance of this finding which is reversible with discontinuation of therapy is unclear.

Further adverse events observed with tirzepatide treatment included sinus tachycardia associated with a concomitant increase from baseline in heart rate of ≥15 beats per minute (4.6%–10.0% *vs*. 4.3% with placebo), hypersensitivity, including severe reactions (3.2% *vs*. 1.7%), injection site reactions (3.2% *vs*. 0.4%), and acute gallbladder disease (0.6% *vs*. 0%) (54, 61). Tirzeptide apparently has no intrinsic risk for inducing hypoglycemia; in clinical studies, an increase in hypoglycemia incidence was only observed in tirzepatide-treated patients if they had a comedication with insulin and/or oral insulinotropic agents (sulfonylurea) (61). Hypersensitivity reactions and antibody formation associated with tirzepatide treatment are comparable in frequency as in GLP-1RA therapy (61). The US prescribing information for tirzepatide carries a black box warning for the risk of thyroid C-cell tumors; tirzepatide is contraindicated in individuals with personal or family history of medullary thyroid carcinoma or in patients with multiple endocrine neoplasia type 2 syndromes (54, 61).

## Perspectives and conclusions

Tirzepatide has demonstrated marked and dose-dependent reductions in glycemic parameters in patients with T2D that, according to the present study data, surpass the effects of the GLP-1RA dulaglutide and semaglutide (54, 66, 67, 71). Cardiovascular data on endpoints like MACE-3 or MACE-4 are presently not available yet, but an early meta-analysis demonstrated at least cardiovascular safety in patients with T2D treated with tirzepatide (78, 79). Until the data of the SURPASS-CVOT trial are available, tirzepatide will most likely have a place in the algorithm of T2D therapy for patients with obesity and T2D that have the therapeutic aim to normalize glycemia and decrease body weight. The present recommendations of the American and European Diabetes Associations are currently updating their actual recommendation for the treatment algorithm for T2D (17). The updated version may include a body weight decrease by 15% for obese patients, underlining the necessity for treatment with either a GLP-1RA or tirzepatide to reach this therapeutic goal. In the event that the SURPASS-CV study will demonstrate superiority for treatment with tirzepatide compared to standard therapy, tirzepatide may receive a first-line treatment recommendation. Until then, GLP-1RA with cardiovascular benefits remains the recommended substance class for obese patients with T2D and pre-existing atherosclerotic vascular disease or patients with a very high risk for this condition (17).

Tirzepatide may also become an important pharmacological tool for the treatment of obesity if data from the SURMOUNT-1 trial are confirmed in other clinical studies (82). The body weight loss observed in this study opens perspectives for a pharmacological treatment of obesity as an alternative to various procedures of bariatric surgery.

Present data show, that the dual receptor agonist tirzepatide has exceeded the glucose-lowering and body weight-lowering effects compared to the established GLP-1 RAs (54, 66, 67, 71]. Some explanations for these strong effects come from mechanistic studies investigating the proportions of the respective GLP-1 and GIP receptor agonistic properties. Imbalance towards the GIP receptor, combined with distinct signaling properties at the GLP-1 receptor, together may account for the promising efficacy of tirzepatide as observed in various cell models, isolated islets, and perfused pancreas (86). Additional mechanistic studies will however be necessary to fully understand the molecular action of tirzepatide.

Obesity and T2D are important risk factors for having a more severe course of a COVID-19 disease in the case of a SARS-CoV-2 infection. Data from the European LEOSS registry as well as other data show an additive effect of obesity, diabetes, and hypertension on the risk of mortality, which is higher in young and middle-aged patients (18–55 years). Young and middle-aged adult patients with all three of the above risk parameters had a similar adjusted increased risk of mortality as older (56–75 years) nonobese and metabolically healthy patients (87). The SURPASS-4 study also captured COVID-19-related deaths. In this study, the mortality due to COVID-19 was <1% in the tripeptide arms as well as in the insulin glargine arms, with a numerically lower mortality rate in the tirzepatide group (75). Studies directly characterizing the effects of treatment with tirzepatide in patients with T2D and/or obesity and COVID-19 or long COVID have not been published yet.

Furthermore, increased CRP levels explained part of the elevated risk of COVID-19–related mortality with age, specifically in the absence of obesity and impaired metabolic health. In conclusion, the modifiable risk factors obesity, diabetes, and hypertension increase the risk of COVID-19–related mortality in young and middle-aged patients to the level of risk observed in advanced age.

In summary, tirzepatide is the first dual receptor agonist that was recently approved for the treatment of T2D by the FDA. The efficacy regarding lowering glycemia and body weight is stronger than that of GLP-1RAs while the safety profile and the incidence of adverse events seem comparable. So far, no trials have been published that compare different dual- or triple-receptor agonists with each other. With tirzepatide, there is now one GIP/GLP-1 receptor dual agonist with a promising development that may find a prominent place in the treatment algorithm for the therapy of T2D and possibly also for obesity.

## Author contributions

The author searched the available literature on the topic using PubMed, Embase, clincal.trials.gov and is the sole independent author of this manuscript and its content.

## Conflict of interest

The author has provided advisory services to AstraZeneca, Bayer, Boehringer Ingelheim, Eli Lilly, Merck Sharp & Dohme, and Novo Nordisk, and has received lecture honoraria from Bristol Myers Squibb, Novartis and the above-mentioned companies.

## Publisher’s note

All claims expressed in this article are solely those of the authors and do not necessarily represent those of their affiliated organizations, or those of the publisher, the editors and the reviewers. Any product that may be evaluated in this article, or claim that may be made by its manufacturer, is not guaranteed or endorsed by the publisher.
